# Association Between Eating Window and Longevity: A Population-Based Study

**DOI:** 10.1093/geroni/igaf122.1873

**Published:** 2025-12-31

**Authors:** Samaneh Farsijani, Ziling Mao, Haley Grant, Stephen Kritchevsky, Anne Newman

**Affiliations:** University of Pittsburgh, Pittsburgh, Pennsylvania, United States; University of Pittsburgh, Pittsburgh, Pennsylvania, United States; University of Pittsburgh, Pittsburgh, Pennsylvania, United States; Wake Forest University School of Medicine, Winston-Salem, North Carolina, United States

## Abstract

Time-based diets are popular for their health benefits, but their long-term effects on longevity remain unclear, with evidence largely from short-term trials and animal studies. Therefore, we determined the association between eating window and mortality risk in US adults. We conducted a retrospective cohort study using NHANES 2003–2018 data, linked to mortality records through December 2019. The analysis included 33,052 adults (>19 years) with two complete dietary and mortality records. Dietary data were collected via two 24-hour recalls, defining the eating window as the time between first and last intake of any food/beverage containing >0 calorie within 24 hours. We used survey-weighted Cox regression with Restricted Cubic Spline (RCS) modeling to assess nonlinear associations. Models adjusted for demographics, socioeconomic factors, lifestyle behaviors, health status, BMI, and diet quality. Subgroup analyses were conducted by age, sex, and race/ethnicity. Over eight years, 4,158 (8.9%) all-cause, 1,277 (2.6%) cardiovascular, and 989 (2.2%) cancer deaths occurred. The fully adjusted RCS model showed a U-shaped relationship (p = 0.004), with the lowest mortality risk at ∼11-12 hours/day. Eating windows ≤8 hours/day were linked to ≥ 30% higher all-cause mortality, especially in older adults, and >50% higher cardiovascular mortality in older adults, men, and Whites. Extended eating windows (≥15 hours/day) were associated with 25% higher all-cause mortality, particularly among Whites, driven by cardiovascular mortality. Our study suggests that a moderate eating window (∼11-12 hours/day) is linked to the lowest mortality risk, highlighting the need for personalized dietary recommendations. Long-term studies are essential to confirm causality and underlying mechanisms.

